# Widespread Proteomic Changes Observed in a Cohort of Neonates and Infants Undergoing Cardiopulmonary Bypass

**DOI:** 10.1016/j.jacadv.2025.102100

**Published:** 2025-08-20

**Authors:** Christopher A. Mancuso, Dustin Nash, Eiman A. Ali, Ludmila Khailova, Tanner Lehmann, Benjamin S. Frank, Matthew Stone, Sierra Niemiec, Jelena Klawitter, Jesse A. Davidson

**Affiliations:** a Department of Biostatistics and Informatics, Colorado School of Public Health, Aurora, Colorado, USA; b Department of Pediatrics, University of Colorado School of Medicine, Aurora, Colorado, USA; c Children’s Hospital Colorado Research Institute, Aurora, Colorado, USA; d Department of Surgery, University of Colorado School of Medicine, Aurora, Colorado, USA; e Department of Anesthesiology, University of Colorado School of Medicine, Aurora, Colorado, USA

**Keywords:** cardiac intensive care, cardiac surgery, congenital heart disease, outcomes, vasoactive inotropic score

**Figure:**
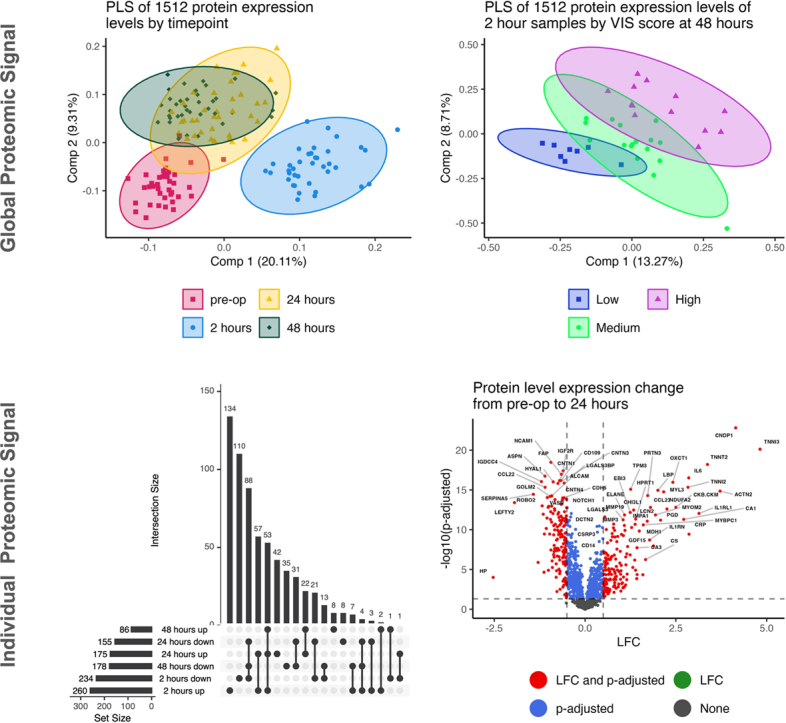

The annual economic burden of complex congenital heart disease is ∼$74 billion, with 10,866 neonates and infants undergoing cardiopulmonary bypass (CPB) in 2023. Although CPB is vital for surgical repair/palliation of congenital heart disease, it is associated with morbidity and mortality through unwanted effects on organ perfusion, metabolism, hemodynamics, and inflammation.^1^ Proteomics offers a powerful way to investigate the molecular mechanisms driving CPB recovery; however, previous works were limited to <30 patients undergoing low-to-medium risk surgeries, used in-house experimental techniques, and were only able to detect <50 significantly dysregulated proteins.^2^, ^3^, ^4^ In this work, we use a highly sensitive commercial protein assay to measure 1,512 circulating proteins in an infant and neonate population, including high-risk surgeries, and find 640 significantly dysregulated proteins and demonstrate the ability to separate patients based on a clinical outcome.

**What is the clinical question being addressed?**

Is a high-coverage proteomic assay able to inform outcomes and provide insights into molecular mechanism driving CPB recovery?

**What is the main finding?**

We find 640 significantly dysregulated proteins and can separate patients based on their VIS score at 48 hours.

We used the SomaScan assay (SomaLogic) to measure circulating proteins from 38 neonates and infants undergoing CPB at Children’s Hospital Colorado. Serum samples (55 μL) were obtained preoperatively after induction of anesthesia and at 2, 24, and 48 hours postoperatively and then frozen at −80 °C. This work is a prespecified secondary analysis of a prospective parent cohort study for which we obtained Institutional Review Board approval and written consent. Global response of the proteomic signal was modeled using partial least squares (PLS) analysis (*mdatools* R package). Significance of individual proteins at each postoperative time point compared to the preoperative samples was determined using paired t-tests and the log2 of the fold change (LFC), with thresholds of an false discovery rate-adjusted *P* value <0.05 and |LFC| >0.5, respectively. Enriched Gene Ontology Biological Processes (GOBPs) were determined for a given set of differentially abundant proteins (DAPs) using the enrichGO function of clusterProfiler, setting the universe of genes to be all proteins measured in the SomaLogic assay. A GOBP term was considered enriched with an false discovery rate adjusted *P* value <0.05.

Patient demographics for this cohort include the following (median, IQR or percentage): age 7 days (6, 70.2), weight 3.52 kg (3.23, 4), 68% male, 31% single ventricle, CPB time 150 min (134, 187), and STAT score 3 (2, 4.75). PLS analysis of the global proteome differed substantially from baseline at all postoperative time points (Figure 1) (R^2^ = 0.53; Q^2^ = 0.50; optimized at 2-components) with the 2 hr time point being the most different, followed by a trend of returning to baseline after 24 hr. We also performed an analysis of how the global proteome discriminates clinical severity using the vasoactive inotropic score (VIS), a metric to quantify cardiovascular support. PLS analysis of the global proteome at 2 hr was able to differentiate patients by their 48 hr VIS score^5^ (low [0-4], medium [5-9], and high [>9]), with good performance metrics (Figure 1) (R^2^ = 0.97; Q^2^ = 0.43; optimized at 4-components). The top ten proteins according to variable importance in projection scores were: 1) increased in higher VIS groups: C3 (complement activation), CASP10 (apoptosis), ENTPD5 (endoplasmic reticular stress), and HMGCS1 (cholesterol formation and endoplasmic reticular stress); and 2) decreased in higher VIS groups: CRLF1 (neuronal survival), DSG2 (cell-cell junction formation), FAP (tissue remodeling and wound healing), PROCR (protein C activation), EPHB6 (thymocyte differentiation), and SELL (cell adhesion).

**Figure 1:**
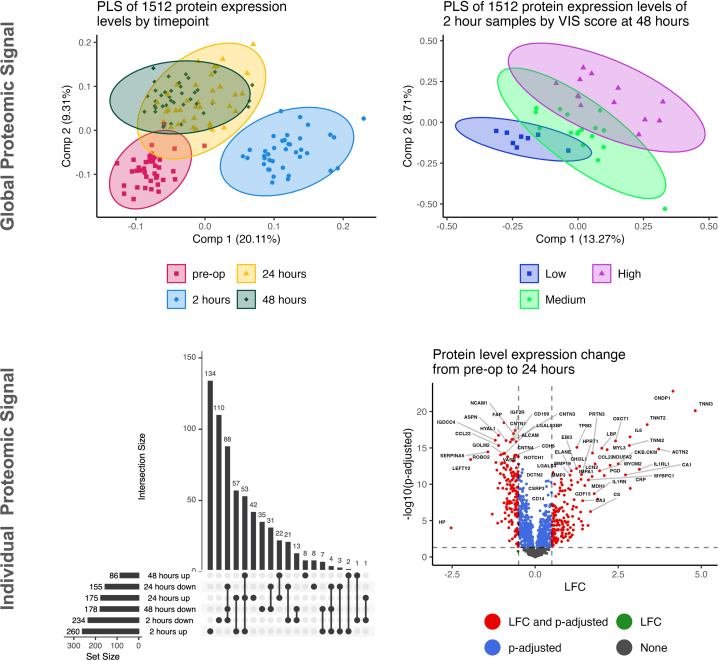
**Global and Individual Proteomic Response of Neonates and Infants Undergoing CBP** Global proteomic response: 2-dimensional visualizations of the first two components from PLS analysis show that the global proteomic signal can separate samples by time point (top-left) and VIS score at 48 hours (top-right). Individual Proteomic Response: Upset plot of DAPs across the time points (bottom-left), and a volcano plot showing wide-spread dysregulation at 24 hours compared to preoperative (bottom-right). CPB = cardiopulmonary bypass; DAP = differentially abundant protein; LFC = log2 of fold change; PLS = partial least squares; VIS = vasoactive inotropic score.

For individual proteins, we found significant dysregulation between preoperative samples compared to the 3 postoperative time points (Figure 1): 2 hrs (260 up, 234 down), 24 hours (175 up, 155 down), and 48 hours (86 up, 178 down). Of the 640 distinct DAPs found across the 3 time points, 145 were dysregulated at all 3 time points, 244 were unique to 2 hours, 50 unique to 24 hours, and 43 unique to 48 hours. Generally, DAPs that were upregulated or downregulated at 2 hours maintained the same directionality at 24 and 48 hours.

Taking a system biology approach, we analyzed the enrichment of DAPs consistently dysregulated across all 3 time points, listing the top 5 proteins per category based on average LFC. For the 53 DAPs upregulated at all 3 time points, we found 8 enriched GOBPs, with most terms related to ribonucleotide biosynthesis (NDUFA2, AK1, HPRT, PKLR, ALDOA) or interactions with symbiont cells (SFTPD, GAPDH, APOL1, ELANE, EEF1A1). For the 88 downregulated DAPs, we found 69 enriched GOBPs. The main categories of enriched terms seen were development of neurons (NOTCH1, CNTN2, ALCAM, CNTN4, and BMP7), development of cardiac structures (NOTCH1, BMP7, NRP2, ERBB3, ENG), kidney tissue and vasculature development (NOTCH1, BMP7, NOTCH3, PDGFA, MMP2), and angiogenesis (NOTCH1, CD160, BMP7, NOTCH3, PDFGA).

We next looked at the 24 hr time point to determine proteomic differences at a point when the acute response to CPB begins to ebb and patients diverge in their clinical courses (Figure 1). The 175 upregulated DAPs at 24 hours led to enrichment of 14 GOBPs that describe cellular detoxification (ALDH1A1, PRDX6, PARK7, MPO, and PRDX1), nucleotide metabolic processes (NDUFA2, PGD, PLA2G2A, MDH1, HPRT1), and hydrogen peroxide metabolic processes (PARK7, MPO, PRDX1, MMP3, PRDX3). The 155 downregulated DAPs enriched 34 GOBPs dealing with neuron development/differentiation (VEGFD, ROBO2, EPHA1, UGDH, L1CAM), chemotaxis (LECT2, VEGFD, CCL22, ROBO2, EPHA1), blood vessel morphogenesis (VEGFD, ROBO2, EPHA1, FAP, IL12 B), and noncanonical Wnt signaling pathway (MYOC, CTHRC1, PTK7, ROR1, FRZB). Several DAPs with the large LFC in magnitude were not associated with any enriched GOBPs but are involved in biologic systems relevant to CPB injury/recovery, including elevated myocardial proteins (TNNI2, TNNI3, ACTN2, TNNT2, CKM) and inflammatory proteins (CNDP1, IL1RL1, CRP, IL6, CA1), and suppressed proteins involved in a range of processes including coagulation, redox reactions, and molecular chaperones (HP, LEFTY2, SERPINA5, GSTA1, IGDCC4, NAAA, SH2D1A, ASPN, TPSAB1, CLUL1).

In conclusion, our pilot study of a large-scale proteomic analysis of infants undergoing CPB identified 640 significantly altered proteins within 48 hours postsurgery, impacting numerous biological processes across multiple anatomical systems. These findings reveal previously unidentified proteomic changes that may inform pertinent clinical outcomes. Large well-powered follow-on proteomic studies are needed to evaluate potential markers, mechanisms, and therapeutic targets aimed at mitigating deleterious postoperative physiology in this high-risk population.

## Funding support and author disclosures

Supported by 10.13039/100000002NIH R01HL156936 (Davidson), 10.13039/100000002NIH K24HL167910 (Davidson), and 10.13039/100000002NIH/10.13039/100006108NCATS Colorado 10.13039/100016220CTSA Grant Number UM1 TR004399. Contents are the authors’ sole responsibility and do not necessarily represent official NIH views. The authors have reported that they have no relationships relevant to the contents of this paper to disclose.
